# Deep Learning for Snake Species Identification to Support Snakebite Treatment: A Comparative Study of Efficient CNN and Vision Transformer Architectures

**DOI:** 10.3390/ani16203298

**Published:** 2026-10-20

**Authors:** Nawanol Theera-Ampornpunt, Panisa Treepong, Phumipat Buaphet

**Affiliations:** College of Computing, Prince of Songkla University, Phuket 83120, Thailand

**Keywords:** snake species identification, fine-grained image classification, knowledge distillation, vision transformer, convolutional neural network, geographic prior, snakebite envenoming, wildlife monitoring

## Abstract

Snakebite envenoming is a neglected tropical disease that affects millions of people each year, and species identification is a critical component in choosing the correct antivenom. In this study, six convolutional neural network and vision transformer architectures are compared for the image-based snake species classification task on the SnakeCLEF 2024 dataset, which contains 1599 snake species observed in 209 countries. We use knowledge distillation to boost accuracy without increasing inference cost, and incorporate country-level geographic location through a closed-form Bayesian fusion of the classifier’s output with location-conditioned species-occurrence priors. Location fusion reduced the classification error rate by over 40% across all architectures, and knowledge distillation provided a further improvement. The best model, CAFormer-S18, obtained an accuracy of 81.71% using both image and country-level location. We also compare the single-image inference latency on a smartphone and a Raspberry Pi 5, revealing that the latency of real-world, on-device deployments is significantly influenced by the interplay of model architecture and inference toolchain. Our top-performing model can infer in about 1 s on both devices, demonstrating that fine-grained species identification of snakes is feasible for real-time use in snakebite treatment and, when combined with an additional object detection stage, in wildlife monitoring.

## Simple Summary

Snakebites injure millions of people every year, and giving the right treatment often depends on knowing which snake species caused the bite. However, the snake is rarely available for examination, and experts who can identify snakes are scarce where bites happen most. This study tested whether a computer model can learn to recognize snake species from a photograph, using a large public collection of snake pictures covering over 1500 species from around the world. We compared several image-recognition models and found that accuracy improved greatly when the model also used the country where the photo was taken, since most countries have only a small number of possible snake species. We also used a training method where one model teaches another, improving accuracy without slowing the final model down. The best model correctly identified the species about eight times out of ten, and could produce an answer in about one second on an ordinary smartphone or a small, low-cost computer. These results show that an affordable, fast, and reasonably accurate tool for identifying snakes from photographs is achievable, which could help guide snakebite treatment and support wildlife monitoring in places with limited access to specialist knowledge.

## 1. Introduction

Snakebite envenoming is a major, longstanding public health problem that disproportionately affects some of the poorest and most remote populations in the world. The World Health Organization (WHO) estimates that approximately 5.4 million people are bitten by snakes annually, with 1.8–2.7 million envenomings, 81,000–138,000 deaths and approximately three times as many permanent disabilities and amputations [1,2]. The majority of these incidents are in low and middle-income countries, especially in rural South Asia, Southeast Asia and sub-Saharan Africa where access to healthcare infrastructure is often limited [3]. Consequently, the WHO formally declared snakebite envenoming a top-priority neglected tropical disease in 2017 and set a strategic goal of halving snakebite-related death and disability by 2030.

A critical, and often overlooked, bottleneck in snakebite treatment is correct and timely identification of the offending species. Antivenom is often species- or genus-specific [4], and administering the wrong antivenom or withholding treatment out of uncertainty can delay effective care, lead to adverse reactions, and worsen clinical outcomes [5,6,7]. However, victims and sometimes even health care providers often cannot identify the species of the snake that caused the bite. The snake may not be caught or killed, physical characteristics described from memory are unreliable, and trained herpetologists who can identify the snake are scarce, particularly in rural and low-resource areas where snakebite is most prevalent [8]. This mismatch between where snakebites occur and where identification expertise is available motivates the need for accessible, automated tools that can assist snakebite victims, first responders, and clinicians in identifying the responsible species quickly and reliably.

Advances in deep learning for fine-grained visual classification offer a promising path toward closing this gap. Modern convolutional neural networks (CNNs) and vision transformers (ViTs) have demonstrated strong performance on fine-grained recognition tasks which require distinguishing visually similar categories, such as identifying snake species from body shape, scale pattern, and color [9]. As a smartphone or a low-cost single-board computer is frequently more readily available in remote or resource-limited settings than a herpetologist, an image-based species classification model deployed on such devices could provide near-instantaneous guidance at the point of care, without depending on network connectivity or specialist availability. For this use case, however, high classification accuracy alone is not sufficient; a model must also be efficient enough to run on ordinary, widely available hardware and deliver a result within a clinically acceptable time frame, as treatment decisions in snakebite cases are often time-sensitive.

There is a significant amount of previous work on the use of deep learning for image-based snake species identification, but typically at a smaller scale than the task addressed here. Several studies focus on a single country or region and a limited, hand-selected set of species; for example, classification of 6 species comparing traditional machine learning (k-nearest neighbors, support vector machines, and logistic regression, with dimensionality reduction) with CNNs such as MobileNetV2 and VGG-16 [10]; 9 species native to the Galápagos Islands using Region-based Convolutional Neural Network (R-CNN) detectors [11]; 22 species found in Malaysia using classical machine learning classifiers with hand-crafted visual descriptors [12]; 26 species native to Morocco using VGG19, VGG16, and EfficientNet-B0 [13]; 28 species using DenseNet, MobileNet, and VGG16 [14]; and 45 cherry-picked species using VGG16, ResNet50, MobileNetV2, and DenseNet121, with an object detection stage used to crop the snake from the image prior to classification [15]. Other studies simplify the label space rather than the geographic scope: one uses DenseNet169 to classify snake images into only two classes, venomous and non-venomous [16], while another performs only coarse-grained animal-type recognition from camera-trap images, with the four classes being snakes, lizards, toads, and background [17]. The reported accuracy in these studies varies from approximately 72% to 97%, but the numbers are not directly comparable to one another or to the current study because they are computed over far fewer and often more visually distinct classes. Classifying among 6 to 45 species from a single country is a substantially easier task than classifying among the 1599 species spanning the global distribution considered in this work. Furthermore, this line of work has consistently relied on CNN architectures introduced before 2020, without benchmarking newer, more efficient CNN designs, vision transformers, or hybrid architectures, and without utilizing knowledge distillation or considering on-device inference latency, the two deployment-oriented concerns central to this study.

Snake species classification has also advanced through the larger scale SnakeCLEF benchmark challenge, part of the annual LifeCLEF challenge and workshop, which has significantly expanded since its introduction: the 2021 edition provided images across 772 species observed in 188 countries [18], while the 2024 edition, on which this study is based, contains images of 1599 species from 209 countries [19]. Reflecting the importance of geographic context for this task, competitive solutions in past SnakeCLEF editions have incorporated location as an extra input, for instance by jointly training a multimodal network that combines learned image and location embeddings [20]. Unlike these learned multimodal architectures, our location fusion approach uses an image-only classifier’s output with location-based species-occurrence priors through a closed-form Bayesian rule applied after training, avoiding the need to jointly train on image and location data together. None of the SnakeCLEF 2024 participants who submitted a working-note paper incorporated geographical location or knowledge distillation into their classification pipeline, and their reported results are typically limited to a single best-performing configuration rather than a systematic comparison across components [21,22,23,24]. Furthermore, prior work on this and similar large-scale benchmarks has been largely concerned with achieving the highest classification accuracy, for example through large model ensembles or learned metadata fusion, with comparatively little attention given to the practical constraints of on-device deployment. In particular, the relationship between architecture choice, model size, and real-world inference latency on mobile and edge hardware, an essential consideration for a tool designed for point-of-care use, remains underexplored, as does the systematic application of knowledge distillation (KD) [25] to improve accuracy without introducing extra inference overhead.

In addition to snakebite treatment, automated snake species identification also has a valuable secondary application in wildlife monitoring and biodiversity research, such as camera-trap surveys, ecological population studies, and the detection of invasive or protected species [26,27,28]. This use case differs from the snakebite treatment scenario in an important respect: wildlife-monitoring cameras typically cover a wide field of view to maximize the likelihood of capturing an animal, so the snake often occupies only a small part of the frame. This challenge is directly reflected in previous studies on camera-trap imagery: one study performed only coarse-grained, animal-type recognition rather than species-level identification, treating all snakes as a single class [17], while another addressed fine-grained snake classification by first applying an object detection stage to crop the snake from the frame before classification [15]. A species classification model trained on closely cropped, snake-centered images, such as those used in this study, is not well suited to wide-field-of-view camera-trap images directly. Thus, consistent with the latter approach, deploying our model for wildlife monitoring requires pairing it with a separate coarse-grain object detection model responsible for locating and cropping the snake within the wider scene, after which the cropped region is passed to the classification model described in this study.

This study makes the following contributions to the snakebite treatment and wildlife monitoring literature. First, we systematically compare the classification accuracy of six modern CNN and ViT architectures, SwinV2-T, EfficientNetV2-S, TinyViT-21M, EVA-02-S, CAFormer-S18, and ConvNeXt-Small, on the SnakeCLEF 2024 dataset spanning 1599 snake species. Second, we apply knowledge distillation across different teacher-student architecture pairings to improve accuracy without any inference-time computation cost. Third, we incorporate country-level geographic location as an additional, low-cost input feature through a Bayesian fusion of the image classifier’s output with location-conditioned species-occurrence priors, with the assumptions underlying this approach explicitly characterized. Fourth, we assess the trade-off between input image resolution and classification accuracy, providing practical guidance for deployment on hardware with constrained compute resources. Finally, and central to the practical motivation of this work, we empirically measure single-image inference latency on a smartphone and a Raspberry Pi 5 across all six architectures. The results reveal that the real-world latency is highly dependent on the interaction between model architecture and the inference toolchain. This effect is not captured by raw parameter count or theoretical computational cost alone, but it has direct implications for selecting models intended for point-of-care deployment.

The remainder of this paper is organized as follows. Section 2 describes the dataset, the six model architectures, our knowledge distillation and location fusion methodology, and our experimental setup. In Section 3, we report our classification accuracy, ablation studies, training dynamics, class-level analysis, and inference delay measurements on both target devices. In Section 4, we interpret these results in the context of the snakebite treatment and wildlife monitoring literature, discuss the limitations of our approach, and outline directions for future work. Finally, Section 5 summarizes our main findings and their implications for practical, real-world deployment of automated snake species identification.

## 2. Materials and Methods

### 2.1. Overview

**Figure 1:**
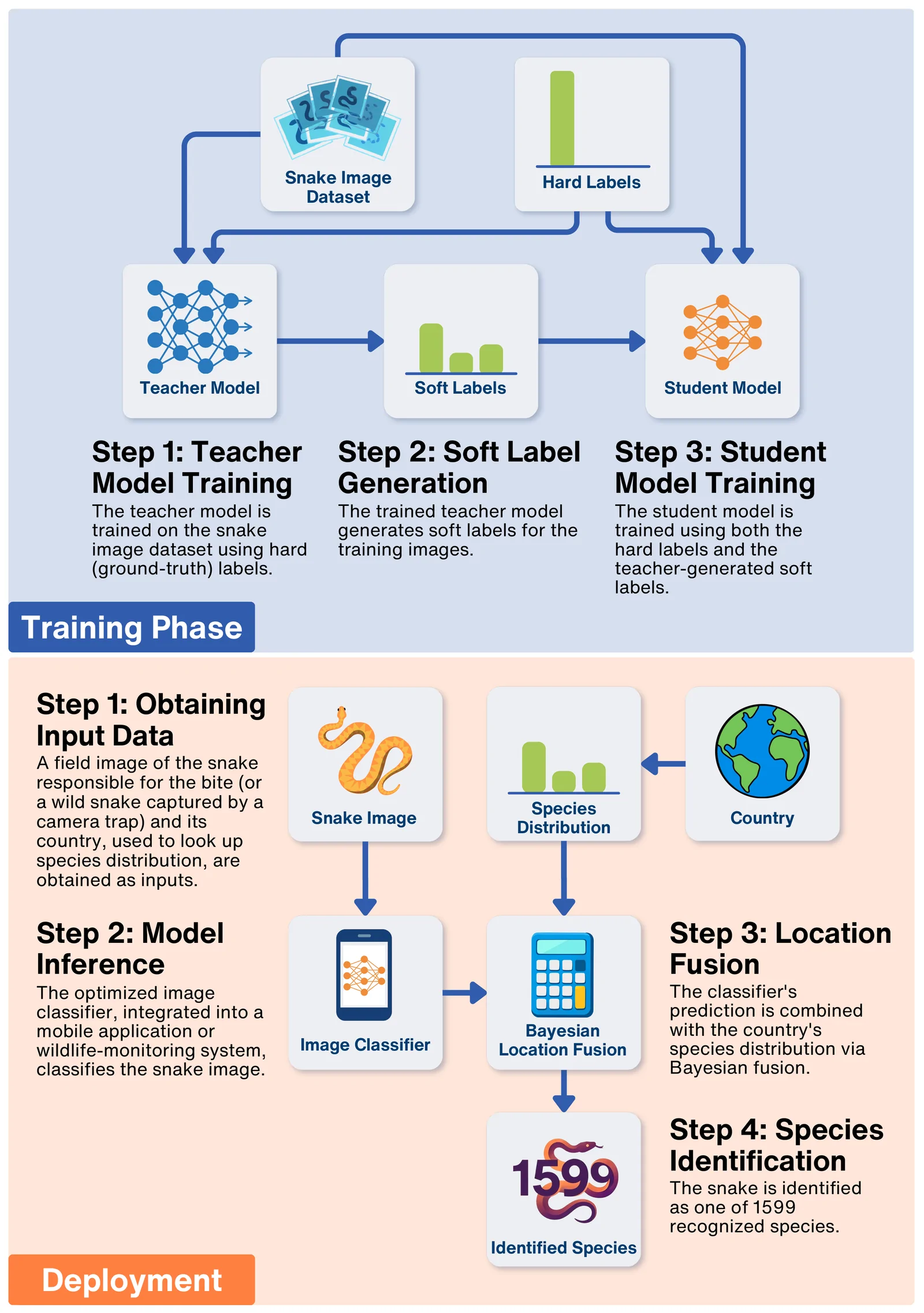
Overview diagram of our snake species classification framework. Arrows indicate the flow of data between components and processing steps.

The overall steps and components of our snake species classification framework are illustrated in Figure 1. In the training phase, we follow the standard KD framework introduced by Hinton et al. [25]. First, the teacher model is trained on the snake image dataset using hard labels. Then, the teacher model is used to generate soft labels of the training images. These soft labels are used alongside the original hard labels and the training images to train the student model. The optimized model can then be deployed as part of a mobile application or wildlife monitoring system to classify snake images in the field, using country-level location and the country’s known species distribution as additional inputs. We will now describe each component and technique in more detail.

### 2.2. Dataset

**Figure 2:**
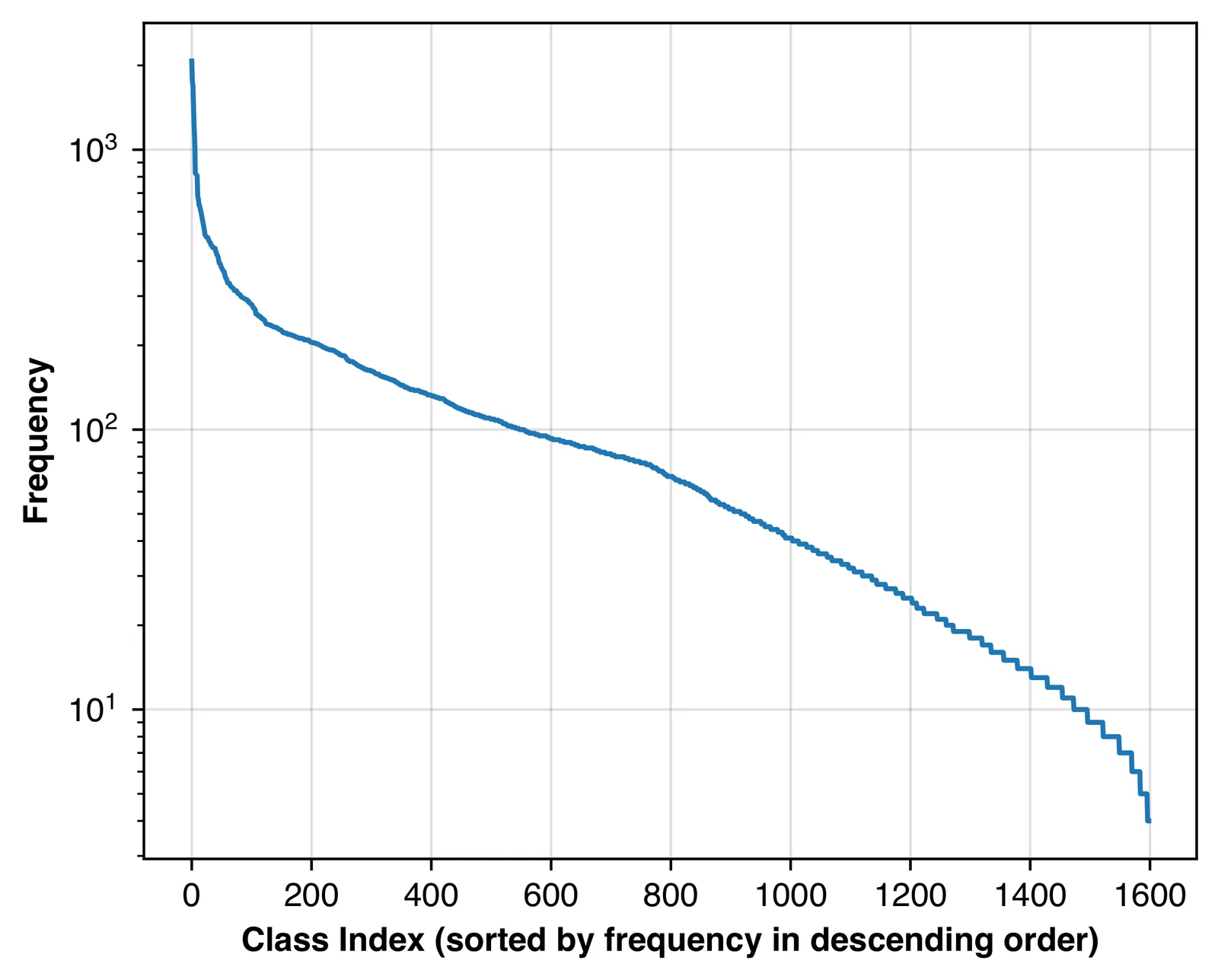
Class distribution of the SnakeCLEF 2024 dataset. Each class corresponds to a unique snake species.

We used the snake image dataset provided as part of the SnakeCLEF 2024 challenge [19], which contains high-quality, preprocessed images of snakes, each labeled with the species by domain experts. The dataset’s organizers publicly released only a training subset and a validation subset; the test subset used to rank competition entries is kept private. To obtain a held-out set for hyperparameter tuning, we further split the provided training subset into our own training and validation subsets. Our training subset was used to fit model parameters, and our validation subset was used to tune hyperparameters. The dataset’s original validation subset, which the model never saw during training or tuning, was then used as our test subset to evaluate the final tuned models. The resulting training:validation:test ratio is 82:9:9. We deliberately did not use these additional training images for rare classes. Many real-world applications of species classification must contend with naturally imbalanced data, since some species are simply photographed far less often than others. By training only on the original, imbalanced class distribution, we demonstrate that our approach remains effective under this realistic constraint, rather than relying on an artificially rebalanced dataset. Full-size images were resized to the required resolution as part of preprocessing. We excluded the 185 species that have training images but no corresponding images in our test subset, since these species can never be evaluated under our protocol. Retaining them would only increase the number of classes the model must distinguish, without being reflected in any reported metric. It also does not make the classification task easier, as none of these species were ever part of our test subset. After this exclusion, 164,898 images of 1599 species remained. The distribution of images across species is highly imbalanced, as illustrated in Figure 2: the number of images per class ranges from 4 to 2074, the top 268 classes account for roughly half of all images in the dataset, and the average and median numbers of images per class are 94.17 and 53.5, respectively. The images were captured in 209 countries spanning every continent except Antarctica. The country-level location metadata provided for each image was used as an additional input feature, as described in Section 2.5.

**Figure 3:**
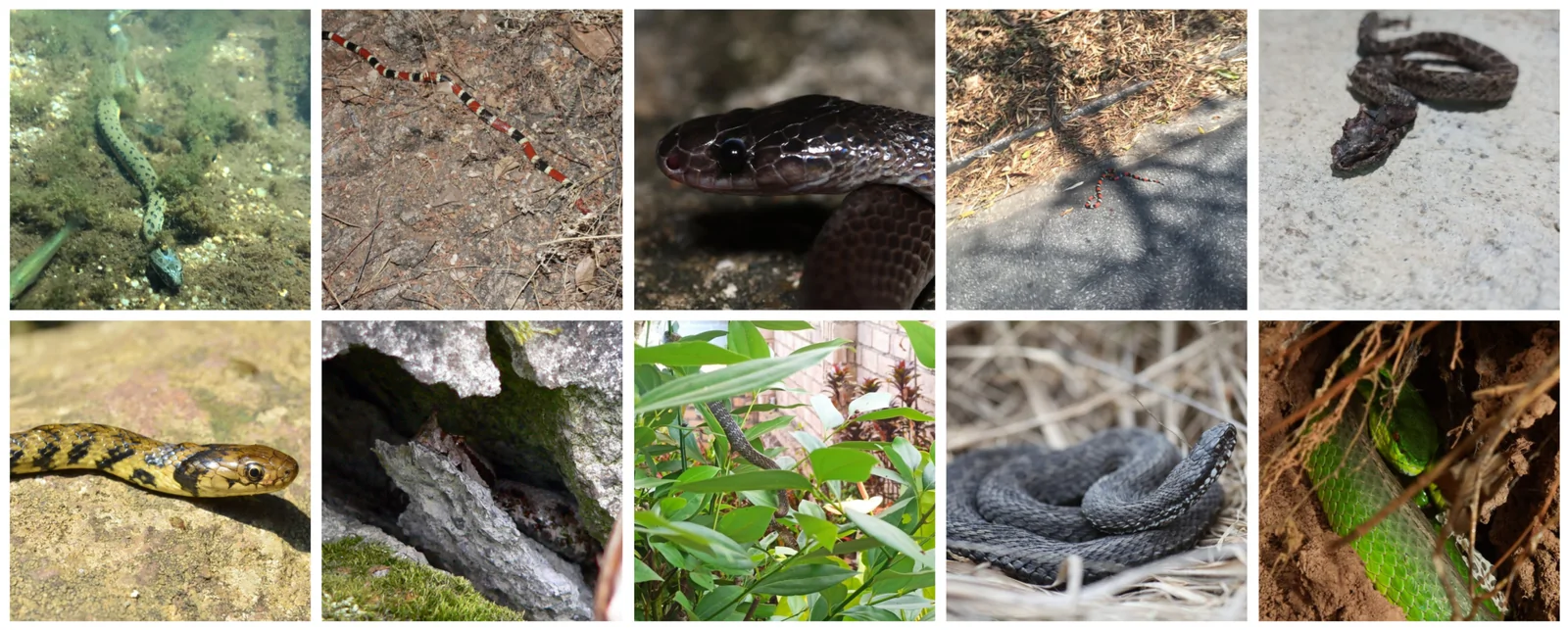
Examples of images in the SnakeCLEF 2024 dataset.

Figure 3 shows example images from the dataset. The field of view, distance, composition, lighting, and surrounding environment vary widely. In many images, only part of the snake’s body is visible, making species identification challenging.

### 2.3. Models

Table 1 lists the six deep-learning architectures used in this study. SwinV2-T and EfficientNetV2-S were included as baselines and were therefore pre-trained only on ImageNet-1K [29,30]. Because ImageNet-1K is a fine-grained dataset that includes many animal classes, including snakes, we selected the remaining four architectures from among those that perform well on this benchmark. To further improve their visual recognition capacity, these four models were additionally pre-trained on the much larger ImageNet-22k dataset before being fine-tuned on ImageNet-1K.

#### 2.3.1. SwinV2-T

This architecture is the tiny variant of the Swin Transformer V2 [31], a hierarchical vision transformer that computes self-attention within local windows and alternates between regular and shifted window partitions across successive blocks to enable cross-window information exchange while keeping computation linear in image size. Compared to the original Swin Transformer, it replaces the learned, absolute relative-position-bias table with a log-spaced continuous position bias and adopts residual post-normalization together with scaled cosine attention. These changes improve training stability and allow more effective transfer across pre-training and fine-tuning resolutions.

#### 2.3.2. EfficientNetV2-S

The EfficientNetV2 [32] family of convolutional networks was obtained through the training-aware neural architecture search jointly optimizing accuracy, parameter efficiency, and training speed. Its early stages use Fused-MBConv blocks, which merge the depthwise and pointwise convolutions of a standard Mobile Inverted Bottleneck Convolution (MBConv) block into a single regular convolution for better hardware utilization at high resolution, while its later stages retain conventional MBConv blocks. The network is also trained with a progressive learning schedule that gradually increases image size and regularization strength during training.

#### 2.3.3. TinyViT-21M

TinyViT [33] is a compact hierarchical vision transformer designed to achieve strong accuracy at small parameter counts through a fast pre-training distillation scheme, in which sparse soft labels from a large teacher model pre-trained on ImageNet-22k are precomputed and stored, allowing large-scale distillation without repeatedly running the teacher during student training. Architecturally, it combines an early convolutional stage with later window-based self-attention stages, and its per-stage width and depth were obtained through a systematic contraction of a larger model family to maximize accuracy under a small parameter budget.

#### 2.3.4. EVA-02-S

This network is the small variant of EVA-02 [34], a plain vision transformer pre-trained with masked image modeling that reconstructs the feature representation of a stronger EVA-CLIP teacher rather than raw pixels. Its transformer blocks incorporate several design choices adapted from recent large language models, including SwiGLU activations in place of a standard feed-forward network, rotary position embeddings for improved extrapolation across image resolutions, and sub-LayerNorm placement for improved training stability, allowing it to reach high accuracy with comparatively few parameters.

#### 2.3.5. CAFormer-S18

CAFormer [35] belongs to the MetaFormer family of architectures, which generalize the transformer block into a token-mixing module followed by a channel Multi-Layer Perceptron (MLP). CAFormer uses convolutional token mixers in its first two, higher-resolution stages for efficient local feature extraction and standard self-attention token mixers in its last two, lower-resolution stages for global context modeling, combining the efficiency of convolution with the long-range modeling capacity of attention. The S18 designation indicates the small-sized variant with 18 total blocks across its four stages.

#### 2.3.6. ConvNeXt-Small

ConvNeXt [36] is a purely convolutional architecture that systematically incorporates design choices popularized by hierarchical vision transformers, such as Swin, into a ResNet-based backbone, without introducing any attention mechanism. Relative to a conventional ResNet, ConvNeXt adopts larger 7 × 7 depthwise convolution kernels, an inverted bottleneck block modeled after transformer feed-forward layers, a patchify stem, and fewer normalization and activation layers, replacing BatchNorm with LayerNorm and Rectified Linear Unit (ReLU) with Gaussian Error Linear Unit (GELU) throughout. These changes allow ConvNeXt to match or exceed the accuracy of comparably sized vision transformers while retaining a pure convolutional structure.

### 2.4. Knowledge Distillation

Knowledge distillation (KD) is a technique introduced by Hinton et al. [25] to improve a model’s classification accuracy by leveraging a teacher model. Neural network training relies on a loss function that guides the model’s parameters toward correctly classifying the training data. Conventionally, this loss is computed against hard ground-truth labels, in which each image belongs to a single class with probability 1. KD introduces an additional loss term that further guides the model toward soft labels, in which each image can belong to multiple classes with different probabilities. These soft labels are produced by a teacher model, which may share the same architecture as the student model or differ from it, using a softmax function with a modified output

(1) $${\hat{y}}_{i}\left(x|T\right)=\frac{\exp\left(\frac{z_{i}\left(x\right)}{T}\right)}{\sum_{j}\exp\left(\frac{z_{j}\left(x\right)}{T}\right)},$$

where ${\hat{y}}_{i}\left(x|T\right)$ is the probability the teacher assigns to class *i* for input image x, $z_{i}\left(x\right)$ is the teacher’s output logit for class *i*, and *T* is a temperature hyperparameter. Setting T≥1 produces a softer probability distribution, which is necessary for effective KD. In this study, we trained the student model using the KD loss

(2) $$l\left(x|T\right)=-T^{2}\sum_{i}{\hat{y}}_{i}\left(x|T\right)\log y_{i}\left(x|T\right)-\sum_{i}{\bar{y}}_{i}\log y_{i}\left(x|1\right),$$

where l(x∣T) is the loss value for image x, $y_{i}\left(x|T\right)$ is the student’s temperature-scaled predicted probability for class *i*, and ${\bar{y}}_{i}$ is the hard ground-truth label for class *i*. The $T^{2}$ coefficient on the first term counteracts the effect of temperature scaling on the gradient magnitude.

Intuitively, KD is effective because soft targets convey information about how the patterns of different classes relate to one another, helping the student model generalize better and converge faster than it would with hard labels alone. Although larger architectures are commonly used as teacher models, architectures of comparable size can also be used effectively, as we demonstrate in this study.

KD affects only the training process: once the student model has finished training, the teacher model is discarded. Consequently, using KD does not increase the computational requirements of the classification model at inference time. Training itself is computationally intensive but can be performed offline, without strict time constraints, using powerful hardware or cloud infrastructure.

### 2.5. Location Incorporation

Fine-grained snake species identification is challenging because of the large number of species and the similarity in body shape and scale patterns among them. For most practical use cases, including snakebite treatment and wildlife monitoring, the country-level location is generally easy to obtain. This location information can substantially aid species classification, because any given country contains only a small subset of all known species. In this study, we used only country-level location, since precise geographic coordinates are more difficult to obtain in practice and are not available in our dataset. To incorporate location into the classification process while keeping the model architecture simple, we adopted the key assumption that image and location are conditionally independent given the species:

P(X,L∣Y)=P(X∣Y)P(L∣Y),

where X is the image, *L* is the country-level location of the snake, and *Y* is the snake’s species.

The output probability that the snake in an image belongs to species *y*, given both the image and the location, is computed as:

(3) $$\begin{array}{cc} P(Y=y|X=x,L=l) & =\frac{P\left(Y=y|X=x\right)\cdot \frac{P(Y=y|L=l)}{P(Y=y)}}{\sum_{y^{\prime }}P\left(Y=y^{\prime }|X=x\right)\cdot \frac{P(Y=y^{\prime }|L=l)}{P(Y=y^{\prime })}}. \end{array}$$

Here, P(Y=y∣X=x) is the prediction of the image-only model, P(Y=y∣L=l) is the probability of species *y* occurring at a given country, and P(Y=y) is the global probability of species *y*. The mathematical derivation of Equation (3) is given in Appendix A.

This formulation is convenient because it does not require training multiple models or a multimodal model that jointly takes an image and a location as input. In this work, we estimated P(Y=y∣L) and P(Y=y) from species counts in the dataset. If more accurate distribution data become available, they can be substituted directly, both for this fusion step and for class weighting during model training and evaluation. Consequently, only an image-only classification model and a table of species distributions by country and globally are required.

Our formulation relies on several simplifying assumptions that warrant discussion. First, the conditional independence of image and location given the species can be violated in practice. For example, location can affect the background fauna and lighting conditions captured in an image. As a result, the fused probability is only an approximation of the true posterior. Second, although the model’s output sums to 1, it may not constitute a well-calibrated probability distribution. Neural networks tend to be overconfident, assigning probabilities close to 1 to the top-predicted class. KD helps alleviate this tendency to some extent, since the student model is also trained to match the teacher’s soft targets, which discourages overconfidence. Third, if the underlying distribution data are not comprehensive, some species may be unreported for a given location. In such cases, the fused model will never predict that species for that location, regardless of how well it recognizes the corresponding visual patterns. This issue did not arise in our experiments, since our distribution estimates were derived directly from the training dataset, which has a similar distribution to the test subset. In settings where the issue does arise, a standard remedy is Laplace smoothing, in which a small constant is added to each species’ count to prevent zero probabilities. We did not apply this smoothing in our experiments.

Our dataset’s metadata specifies the country in which each image was taken. However, location is listed as unknown for roughly 5% of images. For these images, we reverted to the model’s raw image-only prediction.

### 2.6. Experimental Setup

All models were first pre-trained as specified in Table 1 and then adapted to our task through transfer learning, replacing the original classification layer with a 1599-node output layer corresponding to the snake species classes. A dropout layer was added immediately before the output layer for models that did not already include one. Each model then underwent a two-stage training procedure: a transfer learning step followed by a fine-tuning step. In the transfer learning step, only the output layer was trained while all other layers remained frozen. In the fine-tuning step, all layers were trained using a lower learning rate, allowing the model to adapt its visual feature representations to the snake classification task. Training hyperparameters are listed in Table 2. Learning rates were tuned independently for each run. We tuned the maximum learning rate using a greedy local search: starting from an initial value of 0.01, we repeatedly multiplied or divided it by 1.5, retaining whichever direction improved validation accuracy, and stopped once no further change improved it. The best value found was then used as the maximum learning rate. Cosine decay schedule included a one-epoch linear warm-up starting at 0.05 of the maximum learning rate, and decayed to a minimum learning rate of 0. When the cyclical learning rate (CLR) triangular policy was used instead, the initial learning rate was set to 0.05 of the maximum learning rate.

We trained and evaluated all models using PyTorch 2.12.0 using a single NVIDIA RTX 5090 graphics processing unit (GPU) (NVIDIA Corporation, Santa Clara, CA, USA). To perform model benchmarks on the smartphone, we deployed the models using Executorch 1.3.1 [39] with the XNNPACK backend [40]. Model benchmarks on the Raspberry Pi 5 (Raspberry Pi Holdings PLC, Cambridge, UK) were performed using PyTorch 2.12.0.

### 2.7. Evaluation Metric

Models were evaluated using classification accuracy, defined as the number of correctly classified images divided by the total number of images. We report accuracy improvement through the percentage reduction of error rate, defined as 1−accuracy, because it reflects the scale of improvement better than the absolute or relative change in the accuracy values. Each setup was repeated three times, and the average metric value was reported. A statistical significance test was performed using one-tailed Welch’s *t*-test.

We evaluated our models on SnakeCLEF 2024’s publicly released validation subset, repurposed as our test subset, rather than on the private test subset used to rank official competition entries. Consequently, our reported metrics are not directly comparable to the leaderboard results. Furthermore, the competition instead used metrics with an asymmetric penalty, under which mistaking a venomous snake for a harmless one is penalized more heavily than the reverse mistake. To offer a comparable perspective, we additionally report both types of misclassification rate for our top-performing model.

## 3. Results

### 3.1. Model Comparison

We first compare the six architectures’ snake classification accuracy across four setups. The models were first trained standalone using only images as input. We then allowed the models to use country-level location as an additional input. To further improve accuracy, we trained the models using KD. The results are presented in Table 3.

Without KD, TinyViT-21M achieved the highest accuracy regardless of whether location was used as an additional input (*p*-value ≤ 0.012 for image only and *p*-value ≤ 0.0019 for image and location), with the remaining models trailing well behind. Based on this result, we selected TinyViT-21M as the teacher model for the KD setup. With KD, CAFormer-S18 achieved the highest accuracy (*p*-value ≤ 0.0040 for image only and *p*-value ≤ 0.011 for image and location), followed closely by the self-distilled TinyViT-21M. This result suggests that pairing a teacher and student model of different architectures may be more effective than having a single model iteratively improve itself through self-distillation. In every setup, EfficientNetV2-S and SwinV2-T achieved the lowest accuracy, which is expected given that both were pre-trained only on the smaller ImageNet-1K dataset, unlike the other four architectures, which additionally benefited from ImageNet-22k pre-training.

Incorporating country-level location as an additional input feature significantly boosts classification accuracy (*p*-value ≤ 0.0011), as expected, reducing the error rate by an average of 40.46% without KD and 45.20% with KD. On average, each country in our dataset is home to 17.45 snake species. Weighting this average by the number of images available per country raises it to 95.15. This reduction from the full space of 1599 species to a much smaller, location-conditioned candidate set greatly simplifies the classification task, although the task remains far from trivial even at this reduced scale. These results indicate that even a coarse country-level location is an indispensable source of information, one that improves accuracy more than any single modeling technique or architecture choice evaluated in this study.

Adding country-level location as an input feature frequently changes the model’s predicted species, and this change can either help or harm accuracy. Averaged across architectures, adding the location feature converts an incorrect prediction into a correct one 17% of the time, while converting an already-correct prediction into an incorrect one only 0.43% of the time. This pattern varies little across the architectures tested. The large asymmetry between these two rates shows that the benefit of including location substantially outweighs the small amount of error it introduces. This benefit, however, depends on having access to comprehensive and accurate species distribution data; incomplete or biased distribution data could narrow or reverse this advantage.

### 3.2. Ablation Study

**Figure 4:**
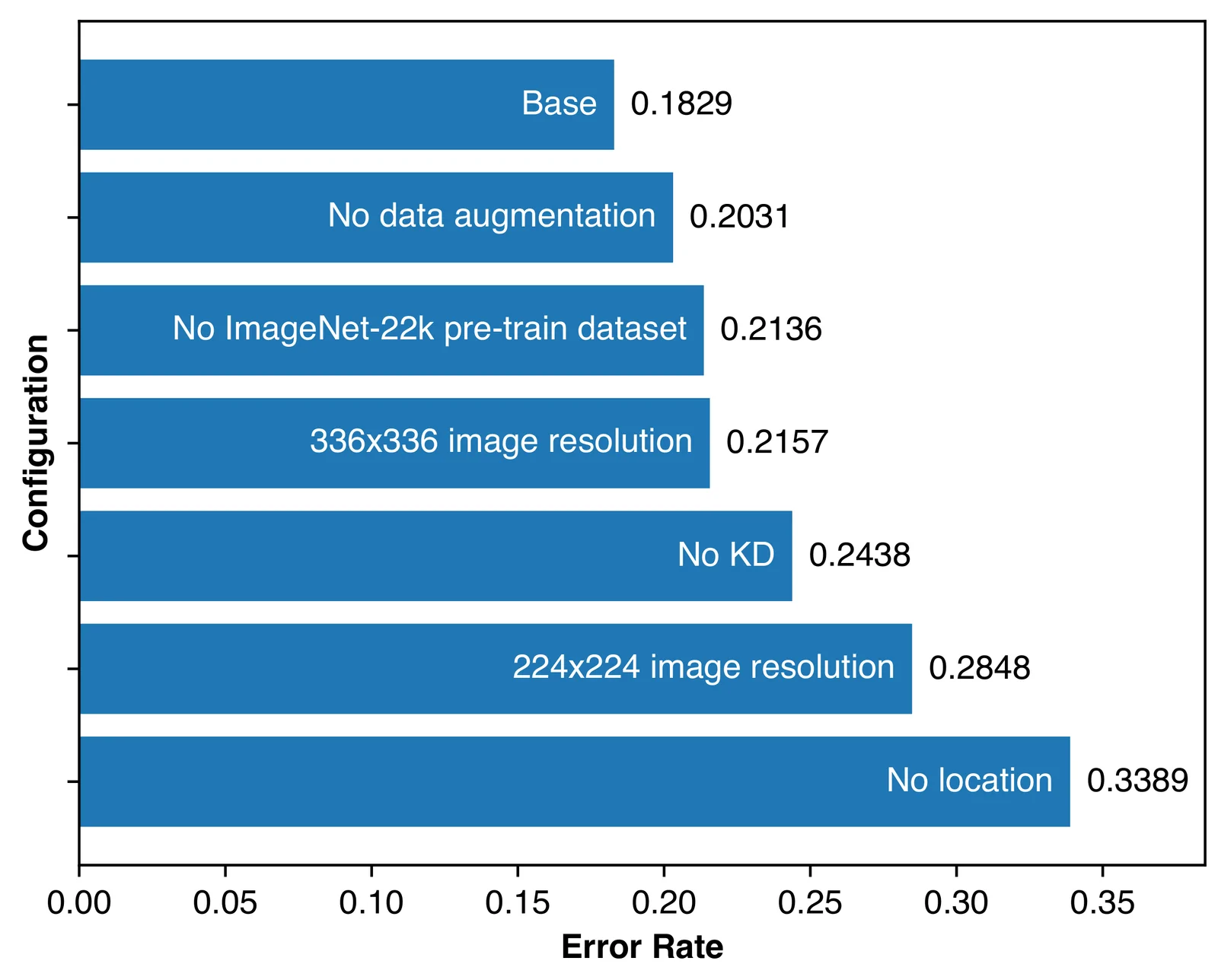
Ablation study results illustrating the effect of removing or modifying a component on classification accuracy. The base image resolution is 448×448 pixels.

In this section, we evaluate how much each configuration aspect or technique affects the final classification accuracy. The components studied are data augmentation, pre-training dataset, KD, image resolution, and location. The experiment is performed using CAFormer-S18 as the student model and TinyViT-21M as the teacher model. The base configuration includes all components. Figure 4 presents the results of the ablation study.

Location is the most important component among those tested, increasing the error rate by 85% when unused. Reducing the model’s input image resolution from 448 × 448 pixels to 336 × 336 pixels increases the error rate slightly, by 18%, while the 224 × 224-pixel resolution produces a significantly higher error rate, by 56%. Removing KD increases the error rate by 33%. Excluding ImageNet-22k pre-training and data augmentation increase the error rate only slightly, by 17% and 11%, respectively. The differences were statistically significant for all components (*p*-value ≤ 0.00052).

Subsequent sections present a detailed analysis of the effects of KD and image resolution.

#### 3.2.1. Knowledge Distillation

Table 4 presents the ablation study on knowledge distillation, using TinyViT-21M as the teacher model throughout. KD improves accuracy for all student models (*p*-value ≤ 0.00066). The first notable result is that KD’s effectiveness is limited when the student shares the teacher’s architecture. In contrast, all other student architectures gain similar, larger improvements from KD. This runs counter to the general trend in the literature, where self-distillation is typically beneficial. We hypothesize that because the self-distilled student has the full capacity to mimic its teacher, both models converge on the same biases and blind spots rather than correcting for them, which can amplify overfitting to those biases rather than mitigate it. Because our results are based on a single teacher model, this trend should be confirmed with additional teacher models before being generalized. Nevertheless, self-distilled TinyViT-21M’s accuracy remains only slightly behind the top-performing model, CAFormer-S18, when KD is applied.

#### 3.2.2. Image Resolution

**Figure 5:**
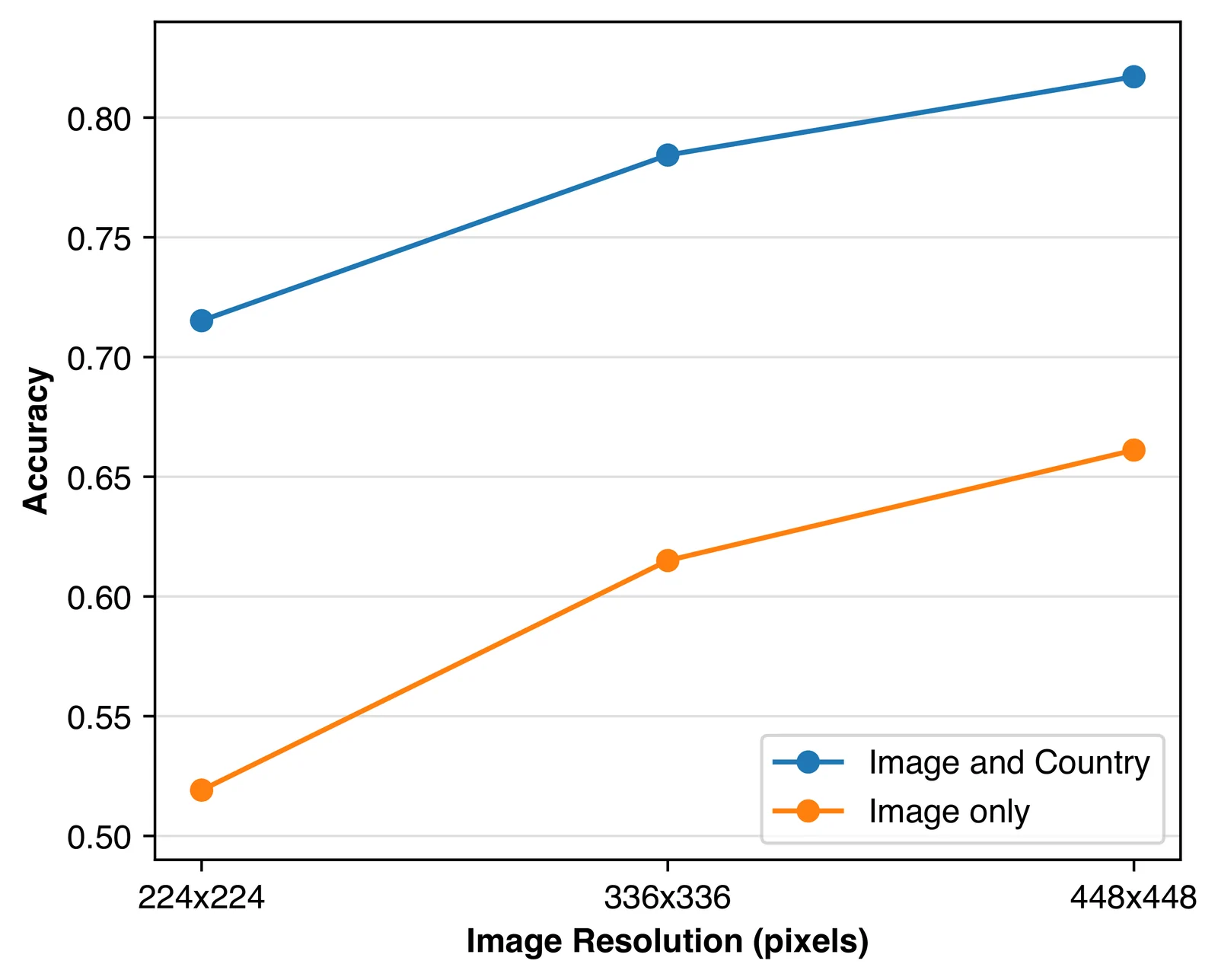
How image resolution affects classification accuracy of CAFormer-S18, the best-performing model, trained with KD, with different sets of input.

Figure 5 depicts the effect of the model’s input image resolution on classification accuracy. Here, we used the best-performing model, CAFormer-S18, trained with KD. We varied the image resolution from 224 × 224 pixels to 336 × 336 pixels and 448 × 448 pixels and experimented with both the image-only and image-and-location prediction configurations.

Higher image resolution allows the model to inspect fine details of the snakes’ scale patterns and body shapes, so it is expected that higher resolution results in higher accuracy. The general trends are similar for both configurations, with the jump from 224 × 224 pixels to 336 × 336 pixels producing a larger improvement than the jump from 336 × 336 pixels to 448 × 448 pixels (*p*-value ≤ 9.6 × 10^−6^ in all cases). Comparing the error reduction from increasing image resolution, the image-and-location setup gains slightly more than the image-only setup. Although there are diminishing returns from increasing image resolution, the curves are still not flat at 448 × 448 pixels, indicating that higher resolutions may yield further gains in classification accuracy. However, higher-resolution images require increased computational resources, generally proportional to the total number of pixels, which is the square of the size of each side in our case. Both the training and inference phases are affected, resulting in a longer wait before an image can be classified, as discussed further in Section 3.5.

### 3.3. Training Dynamics

**Figure 6:**
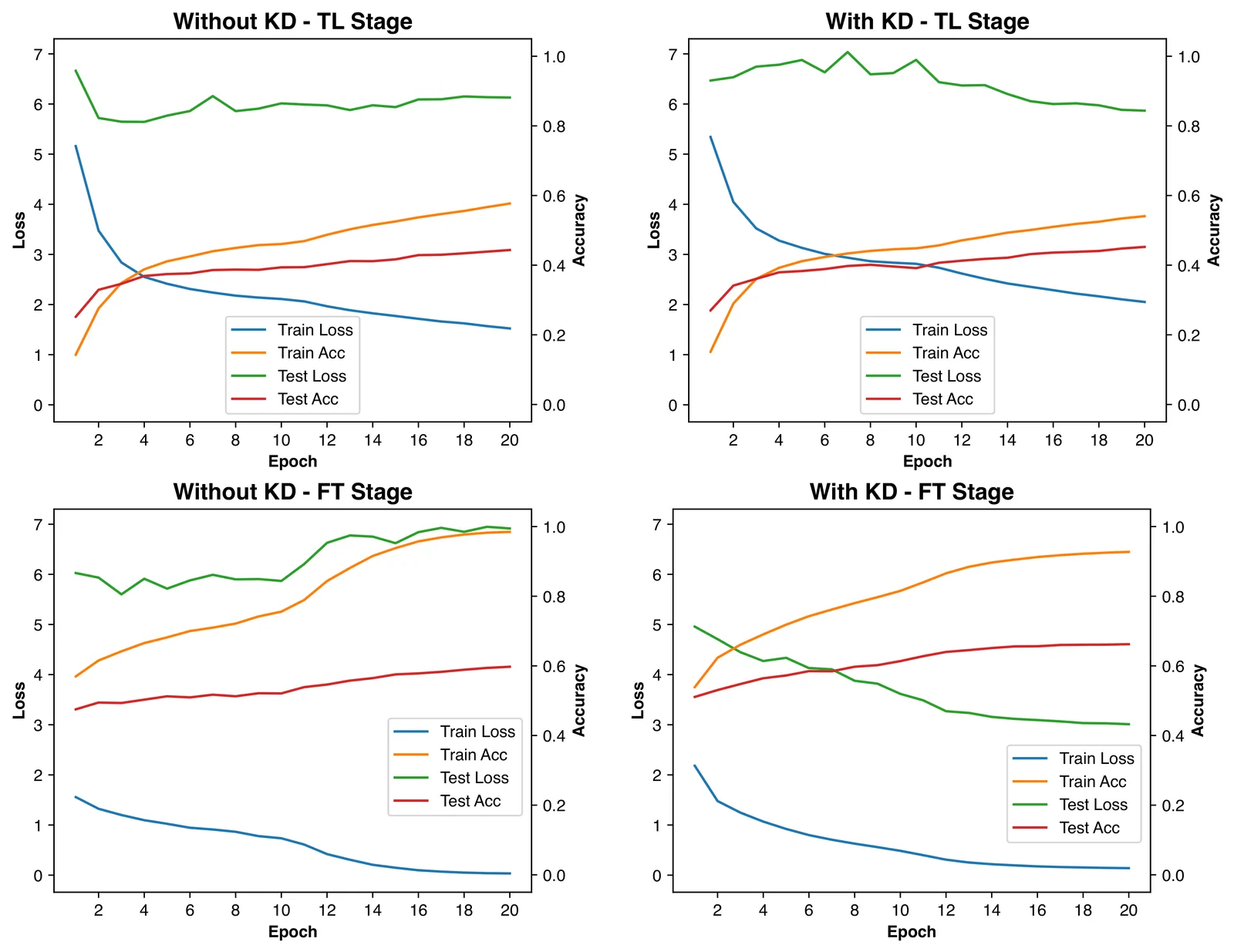
Training curves of CAFormer-S18 during the transfer learning (TL) and fine-tuning (FT) stages, with and without KD. All loss values are computed using standard cross-entropy on the hard labels to allow direct comparison between the two training configurations. The model used only the image as input.

We inspect the training curves of the top model, CAFormer-S18, to understand how KD affects the training process and the resulting accuracy. Because location is incorporated only as a post-hoc fusion step at inference time (Section 2.5) rather than during training, the model itself is always trained using only the image as input. The curves below therefore reflect the training process shared by both the image-only and image-and-location results reported in Table 3. Figure 6 illustrates accuracy and loss on the training and test sets at each epoch.

In the transfer learning stage, the model’s visual feature extraction backbone is frozen and only the output layer is trained, which limits the risk of overfitting. As a result, KD has a negligible effect on the loss and accuracy of either the training or test subset during this stage. In the fine-tuning stage, all of the model’s weights are updated to match the visual patterns in the dataset, which makes overfitting possible. At the first epoch of fine-tuning, loss and accuracy continue from the values reached at the end of the transfer learning stage. Training loss and accuracy improve steadily throughout fine-tuning under both configurations. Without KD, however, test loss stays flat for roughly the first ten epochs and then increases for the remainder of training, while training loss keeps falling. This divergence between training and test loss is the characteristic signature of overfitting: the model continues to fit the training set more closely while its ability to generalize to unseen images degrades. With KD, by contrast, training accuracy saturates around 0.93, since part of the loss is now driven by matching the teacher’s soft-label distribution rather than the hard ground-truth label alone. Importantly, both training and test loss continue to decrease together throughout fine-tuning under KD, with no divergence, and the run ends at a slightly higher test accuracy than the corresponding run without KD. Together, these results suggest that KD enhances generalization primarily by regularizing the fine-tuning stage against overfitting, rather than by improving convergence during transfer learning, where its effect was negligible.

### 3.4. Detailed Analysis

**Figure 7:**
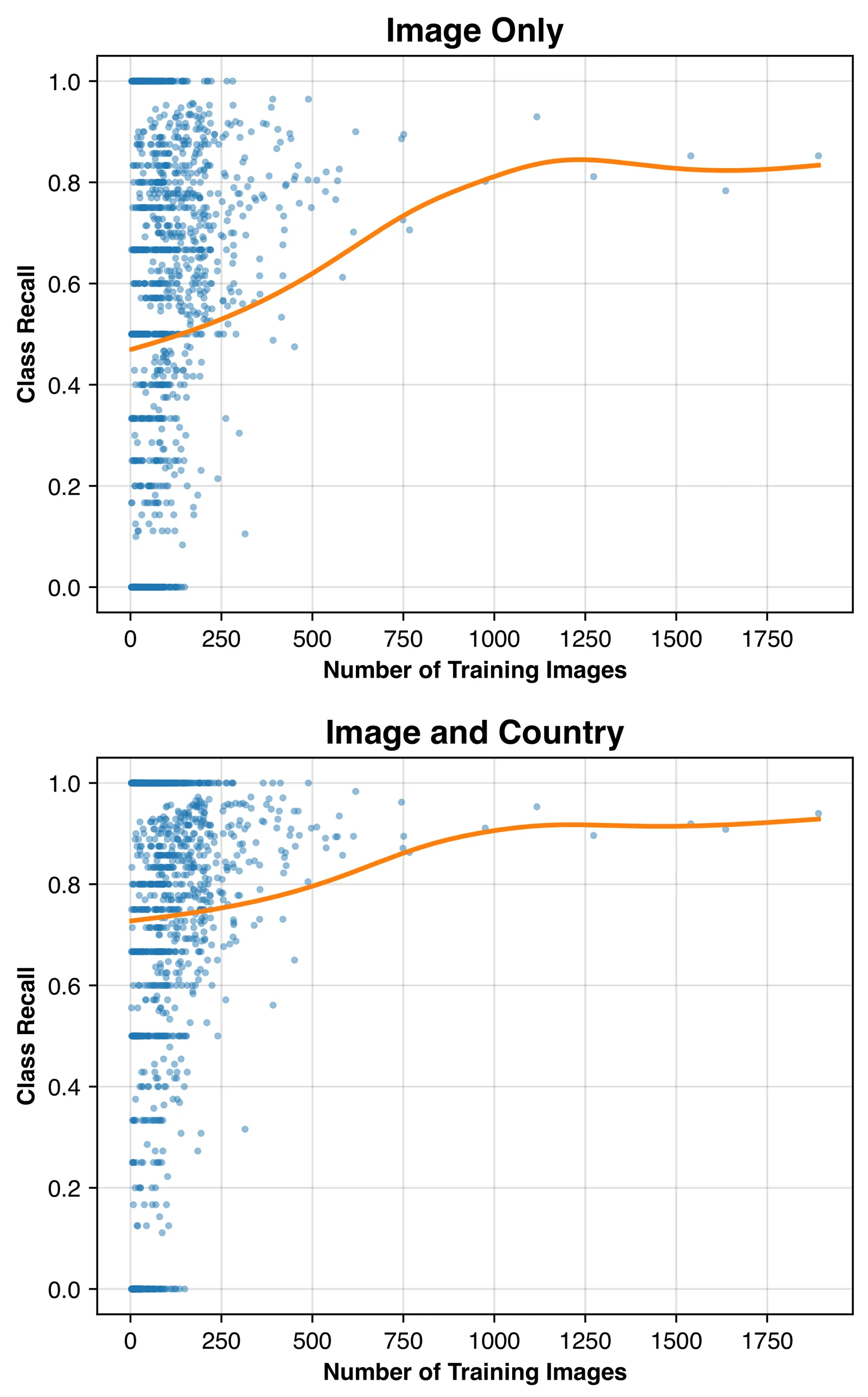
Scatterplots of class recall and the number of training images when only the image is used as input (**top**) and when image and country are used as input (**bottom**). Each dot represents a class (snake species). Orange lines represent trendlines, computed as a Gaussian moving average with a bandwidth of 200.

In this experiment, we take a closer look at the classification accuracy of individual classes to identify factors correlated with high accuracy. Specifically, we inspect class recall, defined as the fraction of images in a class that are correctly classified by CAFormer-S18. Figure 7 illustrates the relationship between the recall of each class and the number of training images in that class. Regardless of whether country-level location was used as input alongside the image, the general trend is that classes with more training images tend to have higher recall. There is only small variability at the top end, while class recall at the bottom end varies wildly, from 0 to 1. This pattern matches the expectations: because the loss function is dominated by classes with more training examples, optimizing for overall accuracy implicitly prioritizes those better-represented classes, and deep-learning models are well known to require large amounts of data to reach optimal per-class accuracy.

Comparing the image-only and image-and-location trendlines shows that incorporating country-level location significantly improves class recall, both for classes with abundant training images and for those with few. We observe that the trendlines reach a class recall of 0.8 at roughly 950 training images in the image-only setting, compared to roughly 500 training images when country is also used. This comparison is intended only as a rough, descriptive illustration of the gap between the two settings, rather than a formal statistical estimate of the exact number of images required.

The model achieved a macro F1 score of 0.7300. Macro F1 is computed as the unweighted average of each class’ individual F1 score, so it gives every class equal weight regardless of how many test images it has, unlike accuracy, which implicitly gives more weight to classes with more test images. Since our training objective was set to optimize accuracy rather than macro F1, it is unsurprising that the macro F1 score is somewhat lower than the accuracy score. Class imbalance also makes it harder to achieve high F1 scores on the rarer classes specifically, which pulls the unweighted average down further. The gap between these two metrics could likely be narrowed by collecting and using more training images for the rarer classes.

The SnakeCLEF 2024 challenge also provides data on whether each snake species is a medically important venomous snake (MIVS). In practice, the real-world cost of a misclassification is likely asymmetric, depending on whether the true species and the predicted species agree on MIVS status: predicting a harmless species for a venomous one is more dangerous than the reverse. CAFormer-S18’s rate of misclassifying a non-MIVS species as a MIVS species is 0.0125, and its rate of misclassifying a MIVS species as a non-MIVS species is 0.0111. These two rates are similar because our training procedure penalizes all misclassifications equally, with no asymmetric cost built in. Cost-sensitive training could instead be used to deliberately lower the more dangerous of these two error rates, at the expense of a higher rate for the other.

### 3.5. Inference Delay

In addition to providing a correct species identification, a snake species identification model needs to give the answer in a reasonable amount of time. The total time needed to go from capturing an image to obtaining a final species prediction involves several steps beyond model inference alone, namely capturing, preprocessing, and, in the wildlife-monitoring scenario, detecting the animal in the frame before classification. To distinguish the model’s own contribution to this total, we define inference delay specifically as the time a model takes to classify an already-preprocessed image. In both the snakebite treatment and wildlife monitoring use cases, it is more convenient and cost-effective to perform inference on mobile devices. Single-image inference delay of deep-learning models on mobile devices may range from sub-second to more than one minute. In this experiment, we measure the inference delay of each model at image resolutions of 224 × 224, 336 × 336, and 448 × 448 pixels. We performed the experiment on the 2021 Samsung Galaxy S21 Ultra 5G smartphone (Samsung, Suwon, Republic of Korea) and the Raspberry Pi 5 device. Raspberry Pi 5 is a credit card-sized, low-power single-board computer well suited for the wildlife monitoring application. We used the central processing unit (CPU) on both devices, since it is supported by all six models, unlike the GPU, whose support varies widely across phone chipsets and is unavailable on the Raspberry Pi 5 entirely. We repeated inference ten times per model and resolution and report the average.

**Figure 8:**
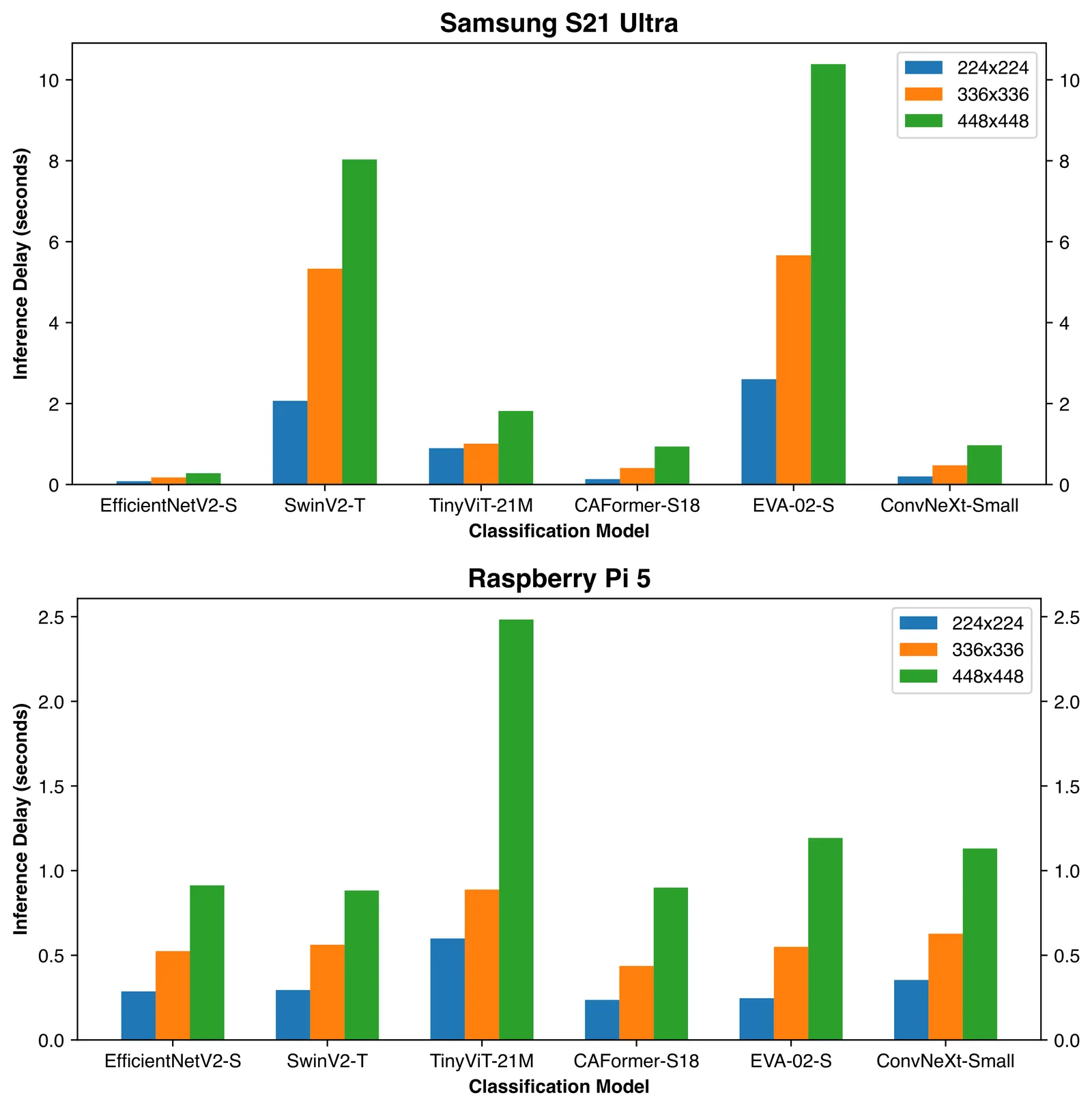
Inference delay of a single image at various image resolutions for each model on Samsung Galaxy S21 Ultra smartphone (**top**) and Raspberry Pi 5 device (**bottom**).

Figure 8 depicts the inference delay of each model on both devices. The most notable and unexpected result is the sharp discrepancy between the smartphone and the Raspberry Pi 5: SwinV2-T and EVA-02-S are 8–10 times slower than the other four architectures on the smartphone, yet perform comparably to them on the Raspberry Pi 5. This discrepancy stems from a difference in inference toolchains rather than an inherent property of the models. On the smartphone, models were exported and executed through ExecuTorch’s XNNPACK delegate [40], which accelerates only a fixed set of operators. Any operator outside this set falls back to an unoptimized, non-vectorized reference kernel, and every transition between an accelerated and a fallback segment of the computation graph incurs additional overhead. SwinV2-T’s shifted-window attention, implemented via cyclic shifts and window partitioning, together with its relative-position-bias lookup, and EVA-02-S’s rotary position embeddings and SwiGLU feed-forward layers, all rely on operators that XNNPACK does not natively support, forcing large portions of these two networks through the slow fallback path at every block. EfficientNetV2-S, by contrast, is the fastest model on the smartphone because it consists almost entirely of depthwise-separable and fused-MBConv convolutions with BatchNorm, both of which are among XNNPACK’s most mature and heavily optimized operators. BatchNorm can additionally be algebraically folded into the preceding convolution’s weights at export time, removing it from the computation graph entirely, whereas the LayerNorm used by the other five architectures cannot be folded in this way and must be computed at every forward pass.

On the Raspberry Pi 5, models were instead run directly with PyTorch and Hugging Face’s inference code in eager execution mode. No ahead-of-time compilation or delegation to a restricted operator set takes place, and every operator, including cyclic shifts, relative-position-bias lookups, rotary embeddings, and SwiGLU, is executed using PyTorch’s native, complete kernel implementations. This removes the fallback penalty that dominates the smartphone results, and SwinV2-T’s and EVA-02-S’s inference delays fall back in line with the other well-supported architectures. Consequently, at 448 × 448-pixel resolution, all models except TinyViT-21M complete inference in 1.2 s or less on the Raspberry Pi 5. TinyViT-21M remains efficient at 336 × 336-pixel resolution and below, but its delay increases sharply at 448 × 448 pixels. This is best explained by its architecture: TinyViT-21M applies windowed self-attention with a fixed window size across several stages, a design tuned for its native 224 × 224-pixel training resolution. At 448 × 448 pixels, the enlarged feature maps must be partitioned into substantially more windows per stage, multiplying the number of small attention and bias-lookup operations that must be executed sequentially. Because eager execution incurs per-operator dispatch overhead with no operator fusion, this increase in operator count, rather than in raw floating point operations (FLOPs), disproportionately slows down the model.

This comparison across devices makes clear that a model’s parameter count and FLOPs provide only a rough estimate of its real-world inference time. The deployment toolchain’s operator coverage, a model’s scalability across image resolutions, and other implementation-specific idiosyncrasies can all have a substantial impact on deployment practicality, independent of the underlying architecture’s theoretical computational cost.

Overall, well-supported models complete single-image inference in under 2 s on the smartphone and under 2.5 s on the Raspberry Pi 5 at 448 × 448-pixel resolution. Mobile devices typically take no more than one second to record, encode, save, and resize an image, independent of the classifier used. In the wildlife-monitoring scenario, an additional object-detection step is also required before classification. Optimizing that detection model, however, falls outside the scope of this study. Taken together, these results demonstrate that our snake species identification system is practical for real-world use on commodity hardware. Even models poorly supported by the current mobile toolchain complete inference in 8–10 s, which remains within a usable range but represents a substantial wait for the user. Our most accurate model, CAFormer-S18, completes inference in roughly one second on both devices and is therefore our recommendation for deployment.

## 4. Discussion

Our results demonstrate that classification accuracy for fine-grained snake species identification can be substantially improved through two complementary, low-cost additions rather than through architecture choice alone. Location fusion nearly halved the misclassification rate across every architecture tested, at essentially no additional inference cost, since it requires only a lookup table of species-by-country frequencies rather than a larger or more complex model. KD provided a further, smaller but consistent accuracy gain, and crucially does so without increasing inference-time computation, since the teacher model is discarded once training is complete. Together, these results suggest that for real-world deployment of fine-grained species classifiers, practitioners may gain more from combining a moderately accurate image classifier with cheap auxiliary signals and training-time techniques than from pursuing ever larger or more sophisticated architectures alone.

The finding that cross-architecture distillation outperforms self-distillation and that pairing dissimilar architectures, a convolution-attention hybrid student with a windowed-attention teacher, achieves the best overall accuracy suggests that architectural diversity between teacher and student plays a meaningful role in KD effectiveness for this task. This is worth further investigation beyond the scope of this study, for example by systematically varying teacher-student architecture pairs or using multiple teachers. At the same time, our class-level recall analysis shows that accuracy gains are not distributed evenly across species: recall for data-scarce classes remains highly variable, ranging from 0 to 1, even with location fusion. For a snakebite treatment use case, this variability is a meaningful limitation, since medically important species are not necessarily among the most frequently photographed in citizen-science datasets such as SnakeCLEF. Data scarcity for a given species does not imply reduced clinical relevance, and a model deployed for snakebite triage should ideally be evaluated and have its confidence calibrated, specifically on medically important species, rather than relying on aggregate accuracy across all 1599 classes.

The accuracy improvement from location fusion should also be interpreted alongside the assumptions underlying our fusion method (Section 2.5). Because the geographic species-distribution table was estimated directly from the training dataset, it inherits the same citizen-science sampling biases as the image data itself: countries or regions with fewer contributors will have sparser, less reliable distribution estimates, independent of the true species richness of that region. Species ranges also change over time, for example due to climate change or habitat loss, so a distribution table estimated from a fixed training set may gradually become outdated. In addition, roughly 5% of images in our dataset lack location metadata, for which our system reverts to an image-only prediction. Extending the model to gracefully use coarser or partial location information, such as continent-level data when country-level data is unavailable, is a natural direction for future work.

Our inference delay results (Section 3.5) show that deployment practicality depends as much on the target inference toolchain as on the underlying model architecture. SwinV2-T and EVA-02-S incurred an order-of-magnitude slowdown specifically under the ExecuTorch/XNNPACK mobile toolchain due to operators unsupported by that delegate, while performing comparably to the other architectures under native, eager-mode PyTorch execution on the Raspberry Pi 5. This has a direct practical implication: architecture selection for on-device deployment should account for the specific inference toolchain to be used in production and should ideally be validated empirically on the target platform rather than estimated from parameter count, FLOPs, or benchmarks obtained on a different toolchain or hardware target. It also suggests that mobile toolchain support for modern vision-transformer operators, such as shifted-window attention and rotary position embeddings, remains an active gap, and that this gap, rather than these architectures’ underlying computational cost, is the primary practical barrier to their mobile deployment.

For the wildlife monitoring use case, the camera must be set up to capture images of a wide area to increase the probability of capturing the target wildlife. Our models are not designed to work with images in which the snake represents only a small portion of the frame. In this case, a separate coarse-grained object detection model must be employed to first detect the general area of the animal, after which a cropped image can be fed into the snake classification model to identify its species.

Several broader limitations of this study should also be noted. First, our evaluation, including the inference delay measurements, was conducted on the curated, expert-verified images of the SnakeCLEF dataset. Real-world snakebite or field-monitoring photographs are often lower quality, partially cropped, or captured under poor lighting, and evaluating model robustness under such conditions is an important direction for future work. Second, our KD experiments used a single teacher architecture, TinyViT-21M. A broader study of teacher-student architecture pairings, or multi-teacher distillation, may yield further accuracy improvements or reveal more general principles for architecture pairing. Finally, our mobile inference delay results are specific to the ExecuTorch/XNNPACK toolchain. Evaluating alternative mobile inference toolchains, such as TensorFlow Lite, ONNX Runtime Mobile, or oneDNN with the Arm Compute Library, may narrow or eliminate the operator-support gap observed for SwinV2-T and EVA-02-S and would strengthen the generality of our deployment recommendations.

## 5. Conclusions

This study demonstrates that accurate, fine-grained snake species identification, spanning 1599 species from the SnakeCLEF 2024 dataset, can be achieved on hardware suitable for real-world snakebite treatment and wildlife monitoring. Incorporating country-level geographic location through a simple, closed-form Bayesian fusion reduced the classification error rate by over 40% across all six architectures tested, while knowledge distillation provided a further, consistent accuracy improvement at no additional inference cost. Our best-performing configuration, CAFormer-S18 trained with knowledge distillation and combined with country-level location, reached 81.71% accuracy while completing single-image inference in roughly one second on both a smartphone and a Raspberry Pi 5. Taken together, these results indicate that combining a moderately sized image classifier with low-cost additional input features and training-time techniques is a practical and effective strategy for deploying fine-grained species classification in resource-limited settings, though further work is needed to improve reliability on data-scarce, medically important species and validate performance on field-realistic imagery.

## Figures and Tables

**Table 1:** Deep-learning architectures used in the study and pre-training datasets used.

Architecture	Type	Parameters	Pre-Training Dataset(s)
SwinV2-T [31]	ViT	28.4 M	ImageNet-1K
EfficientNetV2-S [32]	CNN	20.3 M	ImageNet-1K
TinyViT-21M [33]	Hybrid	21.2 M	ImageNet-22k, ImageNet-1K
EVA-02-S [34]	ViT	22.1 M	ImageNet-22k, ImageNet-1K
CAFormer-S18 [35]	Hybrid	26.3 M	ImageNet-22k, ImageNet-1K
ConvNeXt-Small [36]	CNN	50.2 M	ImageNet-22k, ImageNet-1K

**Table 2:** Training setting and hyperparameters for all models, used in both transfer learning and fine-tuning stages.

Hyperparameter	Value
Image Resolution	448 × 448 pixels
Batch Size	256
Loss Function	KD Loss with inverse-class-frequency reweighting
Optimizer	Stochastic gradient descent with Nesterov momentum
Momentum	0.9
Weight Decay	1 × 10^−5^
Learning Rate Schedule	Best of {cosine decay, CLR triangular policy [37]}
Training Length	20 epochs
Data Augmentation	RandAugment (N=2,M=7) [38]
Dropout Rate	0.2
Gradient Clipping	None
Mixed Precision	BF16 via PyTorch Automatic Mixed Precision
Label Smoothing	None

**Table 3:** Classification accuracy of each architecture. Error reduction rates denote each model’s reduction in misclassification rate due to the inclusion of country-level location as input feature. The best result of each setup is highlighted in bold.

Architecture	Image Only	Image and Location	Error Reduction
Without KD:			
TinyViT-21M	**0.6282**	**0.7845**	42.04%
EVA-02-S	0.5552	0.7335	40.08%
CAFormer-S18	0.5835	0.7562	41.46%
ConvNeXt-Small	0.5595	0.7396	40.88%
EfficientNetV2-S	0.5412	0.7220	39.41%
SwinV2-T	0.4955	0.6917	38.88%
With KD:			
TinyViT-21M	0.6507	0.8125	46.32%
EVA-02-S	0.6325	0.8001	45.61%
CAFormer-S18	**0.6611**	**0.8171**	46.02%
ConvNeXt-Small	0.6452	0.8056	45.20%
EfficientNetV2-S	0.6159	0.7877	44.73%
SwinV2-T	0.5891	0.7672	43.34%

**Table 4:** Effect of KD on classification accuracy. Error reduction rate denotes each model’s reduction in misclassification rate due to KD. The best result of each setup is highlighted in bold.

Architecture	Without KD	with KD	Error Reduction
Image Only:			
TinyViT-21M	**0.6282**	0.6507	6.03%
EVA-02-S	0.5552	0.6325	17.39%
CAFormer-S18	0.5835	**0.6611**	18.64%
ConvNeXt-Small	0.5595	0.6452	19.46%
EfficientNetV2-S	0.5412	0.6159	16.29%
SwinV2-T	0.4955	0.5891	18.54%
Image and Location:			
TinyViT-21M	**0.7845**	0.8125	12.96%
EVA-02-S	0.7335	0.8001	25.02%
CAFormer-S18	0.7562	**0.8171**	24.98%
ConvNeXt-Small	0.7396	0.8056	25.35%
EfficientNetV2-S	0.7220	0.7877	23.63%
SwinV2-T	0.6917	0.7672	24.48%

## Data Availability

The original data presented in the study are openly available at https://www.imageclef.org/node/319 (accessed on 20 May 2026).

## Abbreviations

The following abbreviations are used in this manuscript:

WHO	World Health Organization
CNN	Convolutional neural network
ViT	Vision transformer
R-CNN	Region-based Convolutional Neural Network
KD	Knowledge Distillation
M	Million
MBConv	Mobile Inverted Bottleneck Convolution
MLP	Multi-Layer Perceptron
ReLU	Rectified Linear Unit
GELU	Gaussian Error Linear Unit
exp	Exponential function
log	Logarithm
CLR	Cyclical learning rate
GPU	Graphics processing unit
TL	Transfer learning
FT	Fine-tuning
MIVS	Medically important venomous snake
CPU	Central processing unit
FLOPs	Floating point operations

## Appendix A

For clarity, we omit the instantiated values of X, *L*, and *Y* in the intermediate steps below and write conditional probabilities in terms of the random variables alone. The final result is then restated with all values instantiated, as this is the form used for computation in Equation (3).

Starting from Bayes’ rule, the posterior probability of interest is:

$$P\left(Y|X,L\right)=\frac{P(X,L|Y)P(Y)}{P(X,L)}$$

Applying the conditional independence assumption gives:

(A1) $$P\left(Y|X,L\right)=\frac{P(X|Y)P(L|Y)P(Y)}{P(X,L)}$$

Substituting $P\left(X|Y\right)=\frac{P(Y|X)P(X)}{P(Y)}$ into Equation (A1) gives:

(A2) $$\begin{array}{cc} P(Y|X,L) & =\frac{P(Y|X)P(X)}{P(Y)}\cdot \frac{P(L|Y)P(Y)}{P(X,L)}\\ \; & =P\left(Y|X\right)P\left(L|Y\right)\cdot \frac{P(X)}{P(X,L)} \end{array}$$

Similarly, substituting $P\left(L|Y\right)=\frac{P(Y|L)P(L)}{P(Y)}$ into Equation (A2) gives:

$$\begin{array}{cc} P(Y|X,L) & =P\left(Y|X\right)\cdot \frac{P(Y|L)P(L)}{P(Y)}\cdot \frac{P(X)}{P(X,L)}\\ \; & =P\left(Y|X\right)\cdot \frac{P(Y|L)}{P(Y)}\cdot \frac{P(L)P(X)}{P(X,L)} \end{array}$$

Since $\frac{P(L)P(X)}{P(X,L)}$ does not depend on *y*, it can be treated as a normalization constant, giving:

$$\begin{array}{cc} P(Y=y|X=x,L=l) & =\frac{P\left(Y=y|X=x\right)\cdot \frac{P(Y=y|L=l)}{P(Y=y)}}{\sum_{y^{\prime }}P\left(Y=y^{\prime }|X=x\right)\cdot \frac{P(Y=y^{\prime }|L=l)}{P(Y=y^{\prime })}} \end{array}$$
